# Let the Children Play: Scoping Review on the Implementation and Use of Loose Parts for Promoting Physical Activity Participation

**DOI:** 10.3934/publichealth.2016.4.781

**Published:** 2016-09-26

**Authors:** Natalie E. Houser, Lindsay Roach, Michelle R. Stone, Joan Turner, Sara F.L. Kirk

**Affiliations:** 1 School of Health and Human Performance, Dalhousie University, 6230 South Street, Halifax, NS B3H 4R2, Canada; 2 Department of Child and Youth Study, Mount Saint Vincent University, 166 Bedford Highway, Halifax, NS B3M 2J6, Canada; 3 Healthy Populations Institute, Dalhousie University, PO Box 15000, Halifax, B3H 4R2 and IWK Health Centre, University Avenue, Halifax, NS, Canada

**Keywords:** loose parts, physical activity, active play, outdoor play, unstructured play, children, physical literacy, affordance theory

## Abstract

Active play has become a critical focus in terms of physical activity participation in young children. Unstructured or child-led play offers children the opportunity to interact with the environment in a range of different ways. Unstructured materials, often called loose parts, encourage child-led play, and therefore may also promote physical activity. The purpose of this scoping review was to determine what is currently known about how loose parts may influence physical activity participation. Following a systematic literature search, a total of 16 articles were retrieved, reviewed and categorized according to: (1) types of loose parts; (2) types of play; and (3) types of thinking. We found that there are currently a range of loose parts being used to support play, but the way in which they are implemented varies and there is a lack of clarity around how they might support the development of active outdoor play and physical literacy skills.

## 1. Introduction

Active play is an essential component of children's lives that contributes to physical development as well as cognitive, social and emotional wellbeing [1]. Although there are currently many ways to describe play, active free play can be described as any form of unstructured physical activity participation [2]. Active outdoor play can promote social skills, motor development, and overall physical activity [3]. The importance of active outdoor play is gaining prominence, in part through the release, in 2015, of an active outdoor play position statement [4]. This statement recognizes that “access to active play in nature and outdoors, with its risks, is essential for healthy child development”, and it recommends “increasing children's opportunities for self-directed play outdoors in all settings—at home, at school, in child care, the community and nature” [4]. Not only does play provide ongoing enjoyment, it also has the ability to support a variety of important developmental milestones ranging from movement development to language, conversation, and problem solving abilities [5]. In today's society, characterized by lower physical activity rates and more time spent in sedentary behaviours [3], there is a growing focus on the importance of play to enhance physical activity and movement development, thereby influencing physical literacy. Physical literacy is defined as the motivation, confidence, physical competence, knowledge and understanding to be physically active for life [3]. Previous research regarding loose parts and unstructured play has focused on the implementation of increased structure during play time, even in children as young as 3–5 years old. Research has yet to identify significant improvements in movement development resulting from free play [6],[7]. More recently, research is finding that children may interact with unstructured materials in ways that allow for discovery and engagement in a more physically active way [8], supporting the importance of incorporating loose parts and unstructured play to encourage increased physical activity participation and physical literacy development. Although motor development, the process of acquiring and working on movement patterns and skills, is an important aspect of physical literacy, unstructured and child-directed free-play is essential for various other aspects of children's development and should therefore not be overlooked [1],[9]. Unstructured play is described as child-led play which has no specific outcome or rules in mind, allowing for the child to work on decision-making and discovery on their own [1]. This is different from structured play which has a set outcome in mind and is often adult-led [1].

The idea and relative importance of creative, open-ended play for children's development has been accepted for years [10]. The theory of loose parts, entitled “How NOT to cheat children”, was developed by Simon Nicholson in 1971. Loose parts are defined as materials that are variable, meaning they can be used in more than one way so that children can then experiment and invent through play [10], and these materials can be natural or synthetic. The theory itself arose from two simple factors: a lack of evidence to support the idea that some individuals are born creative and others are not, along with an abundance of evidence supporting the fact that all children love to play and interact with their surroundings [10]. From these observations, the theory of loose parts states simply; “In any environment, both the degree of inventiveness and creativity, and the possibility of discovery, are directly proportional to the number and kind of variables in it” [10]. Not only is unstructured play important, but providing children with the right type of play materials should act to enrich their playing environment, leaving more room for creativity and growth. Object affordance is a theory that refers to how aspects of the environment can offer different opportunities for action or use [11]. These affordances are different for each individual. One child may see a stump as something to balance on, while another child might instead see this same stump as something to climb. This philosophical concept connects the mind and body of the child with characteristics of the environment [11], in this case loose parts. Characteristics of the outdoor environment such as flat surfaces, hills, and trees all afford the opportunity for both physically active and risky play in children [11].

Although the theory of loose parts was developed over 40 years ago, the use of loose parts in practice to support play is unclear, and even less clear is how loose parts might support movement and the development of physical literacy. Through the affordance theory, connections can be made between loose parts and physical activity participation among children [11]. These connections are likely based on the way each child interacts with the loose parts within their environment, but the extent of these connections needs to be better understood. Therefore, the purpose of this paper was to explore the existing knowledge on the theory of loose parts and to determine if and how loose parts are being used to help promote children's unstructured, active free-play.

## 2. Methods

The current review is considered a scoping review, as it focuses primarily on the extent of information available, as opposed to the quality of the articles reviewed [12]. This type of review is useful for exploring the extant literature available on a given topic [12].

**Figure 1.**
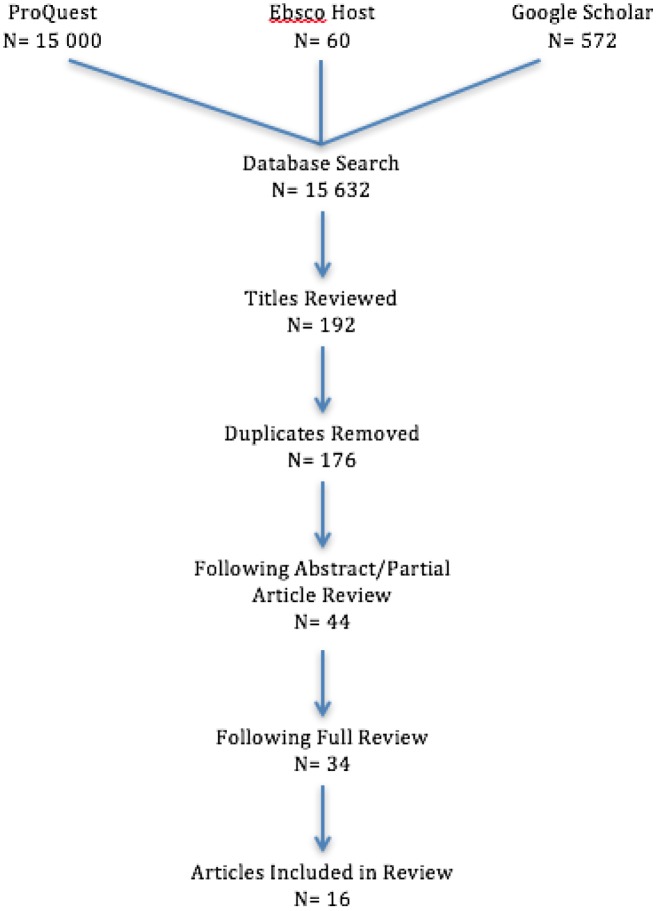
Search Outcome.

To search for relevant scholarly reports related to “loose parts”, a literature review was completed using the following online databases: Canadian Research Index, CBCA Complete, ERIC, Periodicals Archive Online, ProQuest Research Library, Social Services Abstracts, and Sociological Abstracts through the ProQuest search engine along with Academic Search Premier, SPORTDiscus, PsychINFO, PsychArticles, the Teacher Reference Center and CINAHL, searched collectively through EBSCO host. The keywords used were: “loose parts”, “objects”, “plaything”, “toy(s)”, “material”, “natural”, “open ended”, “idiosyncratic”, “informal”, “free”, “outdoor”, “unstructured”, “informal”, “creative”, “explore”, “active”, “imagination”, “creative”, and “play”. The inclusion criteria required that articles be full-text editions from the year 1970-onwards, relating to children or youth. To narrow down initial findings, subjects were tailored to early childhood education, recreation, educational psychology, and teaching education. These criteria were included in order to ensure that the findings were relevant to the purpose of this research. Results provided 60 potentially relevant articles from the EBSCO group and 15,000 from the ProQuest group. An additional search was completed in Google Scholar using key words “loose parts”, “play” and “preschool” which generated a list of 572 potentially relevant articles. The compiled 15,632 articles were then assessed and only those whose titles and keywords that were related to the current understanding of loose parts were included for a final number of 192 relevant citations. Duplicates were then removed resulting in a total of 176 articles for review. See Figure 1 for a breakdown of the search process. Both peer-reviewed and gray literature were included in this review to allow for a more complete view on loose parts materials and how they are being used to encourage physical activity. The gray literature such a magazine articles allowed for a practical view on the research perspectives outlined in the peer-reviewed sources.

Throughout the search a number of set inclusion and exclusion criteria were developed to narrow the search and tailor results to the specific topic of interest. Focus was to be on loose parts use in normally-developing school aged children (12 years and under), specifically on the child's development or use as opposed to teacher development. Work had to be focused on the use of loose parts, not on the design of playgrounds/play settings, or the type of play (e.g. outdoor play, free-play etc.). Finally, those that focused on other learning outcomes such as literary or art-based skill development as opposed to physical activity-related outcomes were excluded. Using these inclusion and exclusion criteria a partial review of titles and abstracts was completed, narrowing the results to 44. Seven thesis and PhD dissertations were included in this list but were not included in the present review, which focused on published data only. The focus of the thesis and PhD dissertations includes the role of nature and outdoors in dramatic play, behaviours with open-ended materials, and role of nature on children's development. The reference lists of the compiled articles were also scanned to check for additional sources that had not been generated during the search. Articles were removed if they did not contain sufficient information to be adequately summarized for the current review. For example, magazine articles comprised solely of lists of materials that could be found around the house and used to stimulate play, were not considered to be loose parts. A final total of 16 articles were included in the current review.

## 3. Results

Table 1 provides a brief summary of each article including the objective, population or target audience, methods, publication type and general findings. From this it is clear that the literature is broad and variable. The articles retrieved could however be categorized according to: (1) types of loose parts; (2) types of play; and (3) types of thinking. The first category relates to the types of loose parts that might be used to stimulate play. The second category relates to the types of play that are encouraged and/or observed through the implementation of loose parts within environments. The third category relates to the different types of thought and the changing definition of the term “loose parts”. Each is described in detail in the following narrative summary.

### 3.1. Types of Loose Parts

There is currently a very broad understanding as to what makes up a loose part, and the terms used to actually define these loose parts are not clearly defined. For example, the term “loose parts” is used interchangeably with “open-ended materials”. Both natural and synthetic materials were included in the lists of loose parts that were described in the reviewed articles. Nicholson originally described loose parts as materials that are variable, meaning they can be used in more than one way so that children can experiment and invent through play [10]. Neill expanded this definition by claiming that loose parts are materials with no set direction that can be used independently or with other materials [13]. They can be natural or synthetic, and with the exception of safety and your own particular environment, have no limitations other than a child's own imagination. A detailed list of examples which contained a large number of loose parts from other reviewed articles [8],[14]–[16], can be seen in Table 2 [13]. The most all-encompassing definition of loose parts was provided by Sutton who defined them as “any collection of fully movable elements that inspire a person to pick up, re-arrange or create new configurations, even realities, one piece or multiple pieces at a time. Loose parts require the hand and mind to work in concert; they are catalysts to inquiry. Loose parts are the flexible edge of an inviting open-ended interactive environment that allows participants to make an imprint of their intention. Experiences with loose parts provide a profound yet playful way for children to form associations between learning and pleasure” [17]. Sutton's description of loose parts aligns with the theory of object affordance described by Little and Sweller, subsequently influencing physical activity participation among children, through their thoughts and movements. The definition of loose parts is versatile and dynamic, fitting for the materials themselves. Ultimately, any object can be used as a tool for play and discovery as long as it is available and safe for the child's age and development.

**Table 1.** Summary of results.

Authors, country, year	Objective/focus	Population	Document type/Methodology	Key points
Nicholson, 1971; USA [10]	Original Theory of Loose Parts—explores supporting research that does not fall in the fields of art, architecture, or planning and uses this to develop a program to promote creativity in educational, recreational and environmental aspects of children's lives.	Target audience: parents and teachers	Scientific peer-reviewed journal: *Landscape Architecture*. No methods specified.	Found research in the fields of community interaction and involvement design along with behavioural planning and design that supports their theory. Discussed the widespread acceptance of the loose parts theory and its impact on curriculum development and environmental education, stressing that the most interesting loose parts can be found in our surroundings. Even found the theory to apply to the design of art galleries and science museums.
Vandenberg, 1981; USA [18]	To determine how quality of play and use of open-ended materials changes in children.	Children ages 4–10 (n = 45), including 24 males and 21 females	Scientific peer-reviewed journal: *The Journal of Psychology* Qualitatively measuring play activity with open-ended materials	Children between the ages of 4 and 10 years old were observed to see how interactions with objects changed as development progressed. It was discovered that the less developed children took part in much more simple forms of play and construction with the loose objects, while more developed children had much more complex idea of play and construction of the objects.
McLoyd, 1983; USA [19]	To observe how various aspects of children's pretend play varies with high (e.g.: dolls, tea sets etc.) vs. low-structure objects (e.g.: pipe cleaners, boxes etc.) Explores the correlation with early child development theories.	Low income, normally developing preschool children aged either 3.5 or 5 years old (n = 36)	Scientific peer-reviewed journal: *Child Development.* Videotaped children playing in bouts of 30 minutes, twice with low-structure and twice with high-structure objects and compared behaviours.	Younger children engaged in significantly more independent pretend play when provided with high-structure materials however the type of material did not affect cooperative play (children playing together) in either age group. High structure objects were found to elicit a larger total frequency of pretend play than low-structure objects. Findings were consistent with El'Konin's (1966) developmental views.
Drew, 2007; USA [14]	To describe the benefits of open-ended play for children.	Target audience: parents and teachers	Magazine article: *Scholastic Parent and Child Magazine* No methods specified	The use of open-ended materials is believed to influence many aspects of a child's life. The open-ended materials referred to include paint, clay, mud, water, blocks, and Styrofoam. It is explained how the ability to play can influence a child's ability to create a meaningful life. There is less pressure on children as there are no rules or goals to open-ended play, which also leads to no possibility of errors, and in turn offering the freedom for children to take part in play however they see fit.
Maxwell, Mitchell & Evans, 2008; USA [5]	To explore how playground equipment and loose parts affects play behaviours and provide empirical evidence that children build spaces when provided with appropriate loose parts.	Preschool aged (3–5 years) children in a child care setting (n = 32)	Scientific peer-reviewed journal: *Children, Youth and Environments* Observed children in a pre-, during, and post-treatment phase to explore differences In play behaviour with the inclusion of loose parts.	No previous work observed loose part effects in an outdoor environment. Play behaviours were observed before and after loose parts were introduced. Inclusion of loose parts to a playground environment increased constructive play behaviours which consequently increased dramatic play activities.
Spencer, 2008; USA [16]	Highlights the lack of un-organized and open-ended play in this generation's children.	Target audience: parents	Online journal magazine: *parenting.com* No methods specified	Describes how play differs for each of the 4 age groups of infants (birth-12 months) toddlers (1–3 years), preschooler's (3–5), & grade school children (5+), and provides suggestions on ways parents can support their play to encourage self-directed learning. Provides examples of common house-hold supplies that can be used as play materials.
Rockwell, 2010; USA [20]	Unpacking Imagination is a loose parts playground idea originally developed in New York	Target audience: parents	Newspaper article: *The New York Times.* No methods specified.	This loose parts playground intervention was developed as an attempt to diminish childhood obesity and screen time in America. Unpacking imagination features a “playground in a box”, made up of foam blocks and shapes intended to encourage children's creativity and play. The intent is that creative play be accessible to all children with this form of playgrounds.
McGonigle & Bownan-Kruhm, 2011; USA [21]	To explain how children's interactions with nature will help form more physically fit and capable individuals.	Target audience: parents	Magazine article: *Natural Life Magazine* No methods specified	This article argues that outdoor play can play a critical role in encouraging physical activity and increased interaction with the natural environment. It is not only motor skills that are the focus with natural play and loose parts, but other aspects such as creativity and socialization. The idea is to create environments where individuals of a range of ages are free to play and create as they wish.
Sutton, 2011. USA [17]	Explored the effects on play behaviour with the inclusion of loose parts to two outdoor learning museum exhibitions.	Observed approximately 50 visitor groups/families interacting in the museum setting.	Scientific peer-reviewed journal: *Children, Youth and Environments* Observed group's preference for location and children's interactions with surroundings. Caregivers completed enjoyment and assessment surveys.	Inclusion of loose parts was found to increase both the amount of time spent in the area and the parent's rating of that area. Furthermore, loose parts increased the incidence of dramatic play while improving children's engagement and understanding of the content in the area. Interestingly, children were reported to use materials that had not be intended as loose parts for play.
Marshall & Dickinson, 2012; USA [22]	To describe how to effectively equip outdoor environments for the use of open-ended materials.	Target audience: parents and teachers	Scientific peer-reviewed journal: *Teaching Young Children* Commentary	Outdoor play spaces offer a wide range of different opportunities when considering the use of open-ended play materials. Outdoor play may include aspects such as loose parts, music, mud, water, and many other features stemming from the indoor play environment. There is a breakdown of how to implement each of these aspects, along with what happens when they are implemented and why.
Ryan et al., 2012; USA [23]	The study aimed to look for common themes related to loose part use and collaboration between populations (children vs. teachers) and across recording techniques (drawings, written records, photos) regarding which proved to be the favorite or most enjoyable.	200+ elementary school children made up the child population and 7 teachers & 1 play specialist designer made up the adult population.	Website: *Western Society for Kinesiology & Wellness* Mixed methods study using the grounded theory method to examine the implementation of loose parts.	Based on the representations formed by the children and their parents, four themes were developed in the study. The themes include; pretend play, gross motor, construction, and enclosed spaces. These themes helped further develop the project as it was taking place, resulting in interaction and engagement among the children.
Mincemoyer, 2013; USA [24]	To provide an overview of existing loose parts and the idea behind the use and implementation of these objects.	Target audience: parents, teachers, and academics	Journal article: Penn State Extension, *Better Kid Care* No methods specified.	The idea of loose parts was originally developed by Nicholson, with the idea of the environment and creativity in mind. This article describes how loose parts can be incorporated into playground, indoor or natural settings. There is also a description of loose parts, which can include anything from balls and hoops, to more natural objects such as rocks and logs. The article determined that children preferred loose parts over more fancy, task specific toys.
Neill, 2013; USA [13]	Explores the theory of loose parts and why loose parts are important to children's play/development. Suggests different materials (natural and synthetic) that can be used as loose parts.	Target audience: parents and teachers	Curriculum Newsletter: *HighScope educational research foundation* No methods specified	This article describes how children prefer to play with open-ended materials, how using loose parts works to develop problem solving skills and the use of imagination in play. Also included is a description of the materials required for a loose parts seminar for teachers and details on how these parts can be adapted for use with children with special needs.
Drew & Nell, 2015; USA [8]	To demonstrate how open-ended play materials can be used and how they influence different groups of individuals.	Preschool children aged 4 and 5 years (n = 15)	Journal article: *Teaching Young Children* No methods specified	A workshop allowing 4- and 5-year-old children to interact with open-ended materials. It was described how this interaction influenced three groups; the children, the teachers, and the families. This article included materials such as wood blocks, rocks, boxes, fabric, foam shapes, along with many others.
Oncu, 2015; Turkey [2]	To explore children's attitudes towards unstructured play materials, were interested in looking at whether the use of recycled objects could be an adequate means of improving creative thinking.	Preschool children (4–6 years of age) from four schools (n = 126)	Scientific peer-reviewed journal: *Education Journal* Children were observed individually, were asked about preferred play materials and were then provided with various loose parts and asked to demonstrate as many ways as possible that they could play with each object.	Determined that most children tended to use the materials in an ordinary way and few used the materials to foster creative play. They found correlations with gender and preference for certain materials (e.g. girls tended to use the napkin more creatively than boys, while boys demonstrated a higher prevalence of creative play with the box, etc.) along with age, where older children tended to participate in more creative play overall.
Szekely, 2015; USA [15]	To highlight playground materials used in outdoor environments, and how they play a role in creative play and teaching.	Target audience: teachers	Scientific peer-reviewed journal: *Art Education*	In areas of Europe, adventure-style playgrounds have been around for some time now, with the intent of encouraging creative play and incorporating it in an artistic sense. It is only more recently that America has adapted a similar, yet more conservative, way of incorporating loose materials into public play spaces, with a similar intent of enhancing children's creativity.

**Table 2.** Detailed list of loose parts including natural and synthetic options [13].

Manufactured	Natural	Location/Season dependent
Recycled tires	Stones	Sea shells
Pallets	Stumps	Beach rocks
Wooden or plastic crates	Logs	Driftwood
Buckets, tubs, laundry baskets	Large branches	Hay bales
Plastic garden pots	Small twigs	Troughs
Boxes	Sand	Old street signs
Gutters	Gravel	Traffic cones
Drain tile	Water	Car parts
PVC pipe	Leaves	Logs
Wood (planks)	Pebbles	Pine cones
Rope	Sunflowers	
Chain	Seeds	
Cardboard rolls or tubes		
Wooden reels		
Plastic bottles		
Landscape netting		
Ice cream tubs		
Fabric, tarps, mesh		
Hoops		
Bricks		
Chalk		

### 3.2. Types of Play

Incorporating loose parts into a play environment opens up the possibility for various types of play. Whether the child chooses to use them for creative, dramatic, exploratory, cooperative, or constructive play, the loose parts are flexible and can be selectively chosen to ensure that they are appropriate for any particular age group, with children being able to use them in ways that are reflective of their own individual development [13].

Maxwell, Mitchell and Evans conducted a two-part study that first observed the type of play behaviours on different playgrounds and then observed the additional effects of adding loose parts to enhance play behaviours in an outdoor setting [5]. The authors explored different types of play, focusing particularly on dramatic vs. constructive play, and how the addition of loose parts would affect each. In their study, dramatic play was defined as play that involves imagination where the child can pretend to be something, or someone else while constructive play is when children create or build objects in a goal oriented manner. While both types of play were considered to be more complex forms of play behavior, authors emphasized the importance of dramatic play for not only cognitive, but social and emotional development as well, claiming that it is the foundation for the development of abstract thought. However, the prevalence of dramatic play is decreased when enclosed spaces are not available, making it less common during outdoor play [5]. Authors hypothesized that providing children with loose parts would function to increase the prevalence of constructive play, and in particular children would build spaces in the outdoor setting that could then be used to increase dramatic play. Furthermore, they believed that children would show a preference for playing in areas where loose parts were provided. Results confirmed these hypotheses and, upon removal of loose parts, both dramatic and constructive play decreased, demonstrating that the addition of loose parts can affect various types of play behaviour [5]. Sutton also observed an increase in dramatic play following the inclusion of loose parts [17]. In particular, increases were noted in environments where the loose parts were suitable for thematic play, enabling children to easily create their own storylines and incorporate these materials appropriately [17].

Drew and Nell supported the idea that loose parts provide the opportunity for a range of different types of play [8]. Through the interaction with loose parts, it was discovered that while children can derive benefit from loose parts environments, teachers and families also experienced similar benefits [8]. Spencer, on the other hand, chose to detail how play differs depending on a child's age [16]. Her magazine article highlighted the appropriate loose parts for each age group, and provided strategies for parents to help encourage age-appropriate, creative play [16].

### 3.3. Types of Thinking

Similar to types of play, some studies focused more on types of thought or thinking that might be stimulated through loose parts play. Convergent thinking is based on logic and a set of rules or guidelines that are followed [2]. Alternatively, divergent thinking is based on an individual's originality of thoughts, leading to creative thinking and creative play [2]. For example, Oncu explored how the inclusion of loose parts would affect divergent and creative thinking [2]. Divergent thinking can be viewed as a type of thought which generally would lead to the expected or “correct” solution to a problem, as opposed to unique or original decisions seen in convergent thinking. It is considered different from creative thinking as while divergent thinking tends to lead to originality, the main component of creativity, you can still think in a divergent manner without being creative. That being said, the ability to think divergently is viewed as an indicator of one's potential for creative thinking [25]. The study used the terms unstructured materials and loose parts interchangeably, which were implemented to explore preschool children's divergent thinking abilities. The use of unstructured materials was thought to be able to foster more flexible thinking as they could be used in a variety of manners. Ultimately the belief was that by improving divergent thinking ability, overall creative thinking ability would also be improved. To test this, 4–6-year-old children were provided with the materials and asked questions about what they could do with them. Unfortunately, results showed that few children were actually able to use the unstructured materials creatively. This may have been due to the study design, where children had to explain how they would use these materials creatively, a task that may have been too advanced for the young children involved. Despite these findings, however, the authors still suggested the use of recycled and open-ended materials to foster creative play [2].

Szekely emphasizes how loose parts in outdoor environments can foster a creative play setting, while also encouraging children's artistic abilities [15]. The idea behind adventure playgrounds is to stray from conventional playground structures, and instead provide children with the prospect to interact freely and creatively with the playground environment [15]. Initially created in Europe, then later incorporated in a similar way in the United States, these playgrounds provide children with the opportunity to choose for themselves what and how to play [15]. Loose parts further this creative opportunity by providing an environment and materials that are open to the child's own interpretation. Instead of playing in an environment designed by adults with highly structured materials, the child decides how each object can be used. There is a significant difference in affordances for physical activity participation in centres where there are more resources, natural elements present and the amount of outdoor spaces [11], influencing how much moderate-to-vigorous physical activity children obtain in their respective centre. This ability to manipulate one's own environment encourages not only creativity but the development of problem solving abilities. On top of the potential for physical and cognitive development, loose parts foster social interactions between children making them share and create imaginative play scenarios together [13].

## 4. Discussion

This review has identified the scope of literature on loose parts materials through the analysis of several published, peer-reviewed studies that focus on the use of loose parts for active outdoor play, along with several magazine articles. The review provides an overview of the potential that loose parts might have as a means to promote physical activity participation and develop physical literacy in children through unstructured play. However, the available evidence is limited, particularly in how active play with loose parts might impact physical literacy. The search strategy used was kept broad, resulting in a similarly broad range of information that then had to be narrowed down manually. Furthermore, the term itself, “loose parts”, is ambiguous and unfortunately, many of the generated articles related in no way to the concept of loose parts for play. Another major limitation is that there was little comparability between the outcome variable that studies were exploring. Some studies were interested in how loose parts affected creativity [2],[15], others were interested in play behaviours [5],[8],[17] and many studies were excluded as they focused on variables such as language or artistic abilities rather than on physical activity or physical literacy.

Neill argues that the implementation of loose parts encourages a range of types of play including creative play, exploratory play, or dramatic play [13]. Loose parts were also discovered to provide a different way of thinking, fostering creativity and exploration of the environment and of movements [2],[15]. Furthermore, Sutton claims that including a large array of loose parts into a play environment can act to broaden children's minds [17]. Consequently, they begin to see the whole environment as a potential loose part to be used to enhance play [17]. It is also important to note that cultural differences may impact the way in which loose parts are used. This is an area that requires more research to determine how cultural differences in play and the use of loose parts may impact physical activity levels of children.

Another limitation, inherent to loose parts, is that there is no way to predict how children will choose to play with them. This makes it challenging to assess how loose parts might support physical activity and physical literacy and reveals an important gap in the literature. While the malleability of the term loose parts is beneficial for providing ample opportunities to incorporate these types of materials into play, it does complicate research into the benefits associated with loose parts. Prior to further exploration on the impact of loose parts, it would be beneficial to agree on a common definition for the term, as well as the specific parts that should be considered “loose parts” for research purposes. A common definition would allow for a mutual understanding of the concept, while also allowing for research in this area to become more focused and comparable. However, as discussed previously, what and how children use various materials for play is very hard to predict and therefore harder to define. While there does seem to be a fair amount of research on the use of loose parts, the quality is quite variable. For example, Oncu provided children with loose parts and then had them explain how each would be used [2]. This specific approach may have been too complex for ages of children involved. Other studies used observation to visually assess how individuals interact with loose parts and also to listen to the types of interaction they provided [5],[17]. Because each study took a slightly different approach and observed different key variables of interest, it is difficult to make comparisons among them.

When school-aged children are given the opportunity for more outdoor free play after school, physical activity participation increases and sedentary behaviours decrease [3]. Although there is evidence suggesting the benefits to outdoor play in relation to physical activity participation, there is currently minimal information on how loose parts play a role in the outdoor play environment. Given the interest in loose parts to promote active outdoor play [3], we found no research that explored how loose parts impact fundamental movement skill development in children. The ways in which loose parts might be used for both free-play and organized games could help to promote improved fundamental movement skills and it would be beneficial to outline some of the possible movement skills that could be targeted through loose parts inclusion. This could also provide a gateway for further research on how the implementation of loose parts influences physical activity levels and enjoyment.

## 5. Conclusion

The existing articles relating to loose parts provide an overview of the loose parts that are being used, how they are used, and the types of thought and play that these materials can encourage. It is clear that the information currently available regarding the implementation and impact of loose parts is both very broad and limited with respect to promoting physical activity and physical literacy. Loose parts may include anything from logs and rocks to ropes and boxes, highlighting that it is both natural and synthetic parts that fit into this category [8],[13]–[16]. Loose parts provide children with unlimited opportunities to use these materials creatively in order to support the type of play of their choosing, therefore encouraging physical activity participation in a range of different situations and environments. Although there is still a lack of knowledge in this area, the current research demonstrates that the implementation of loose parts does have a positive impact on children, as well as their teachers and family members, but the true extent of this influence, particularly in the promotion of physical activity and physical literacy is still unknown.
